# Access to Autism Spectrum Disorder Services for Rural Appalachian Citizens

**DOI:** 10.13023/jah.0201.04

**Published:** 2020-01-26

**Authors:** Angela Scarpa, Laura S. Jensen, Denis Gracanin, Sharon L. Ramey, Angela V. Dahiya, L. Maria Ingram, Jordan Albright, Alyssa J. Gatto, Jen P. Scott, Lisa Ruble

**Affiliations:** Virginia Polytechnic Institute and State University; Virginia Polytechnic Institute and State University; Virginia Polytechnic Institute and State University; Virginia Polytechnic Institute and State University; Virginia Polytechnic Institute and State University; Virginia Polytechnic Institute and State University; Virginia Polytechnic Institute and State University; Virginia Polytechnic Institute and State University; Virginia Polytechnic Institute and State University; University of Kentucky College of Education

**Keywords:** Appalachia, autism, Autism Spectrum Disorder, evidence-based services, rural health, health disparities, barriers to care

## Abstract

**Background:**

Low-resource rural communities face significant challenges regarding availability and adequacy of evidence-based services.

**Purposes:**

With respect to accessing evidence-based services for Autism Spectrum Disorder (ASD), this brief report summarizes needs of rural citizens in the South-Central Appalachian region, an area notable for persistent health disparities.

**Methods:**

A mixed-methods approach was used to collect quantitative and qualitative data during focus groups with 33 service providers and 15 caregivers of children with ASD in rural southwest Virginia.

**Results:**

Results supported the barriers of availability and affordability of ASD services in this region, especially relating to the need for more ASD-trained providers, better coordination and navigation of services, and addition of programs to assist with family financial and emotional stressors. Results also suggested cultural attitudes related to autonomy and trust towards outside professionals that may prevent families from engaging in treatment.

**Implications:**

Relevant policy recommendations are discussed related to provider incentives, insurance coverage, and telehealth. Integration of autism services into already existing systems and multicultural sensitivity of providers are also implicated.

## INTRODUCTION

Autism Spectrum Disorder (ASD) is a neurodevelopmental condition that begins early in life and impacts functioning in multiple domains, including family and peer relationships, academic placement, and employment opportunities.^1^ Autism services cost Americans roughly $2.5 billion per year, with a similar economic burden in the United Kingdom.^2^ Scientists agree that the earlier a child is diagnosed and receives intervention services, the better the child’s prognosis,^3^,^4^ yet multiple barriers to early identification and intervention exist, including rurality.^5^,^6^ Since early identification and intervention improve prognosis and thereby reduce economic burden of an ASD diagnosis, greater policy attention is warranted, especially for ASD services in rural settings.

Low-resource rural communities face significant challenges regarding the availability of evidence-based services. They are often disadvantaged in terms of geographic proximity to care, particularly specialty care, facing challenges such as poverty, inadequate transportation, unemployment, and limited access to information technology, all of which contribute to diminished service utilization.^5^,^7^–^10^ This, in turn, can lead to ASD being overlooked, misdiagnosed, or untreated.

Several studies outside of the U.S. have aimed to identify the barriers to accessing services for ASD from the perspective of families and service providers in rural regions.^11^–^13^ Results of such studies have identified the high cost of ASD treatment, time commitment for treatment, and proximity to service providers as barriers that disproportionally impact families living in these communities.^11^ Additionally, parents of children with developmental disabilities living in rural communities in Australia have reported a lack of access to relevant information about their child’s diagnosis, as well as the types of services that may be available.^12^ When parents are finally able to access and interact with service providers, they report these interactions to be inadequate.^12^ While these findings emphasize many of the challenges associated with accessing services for individuals with ASD while living in rural areas, questions remain with respect to the unique experience of rural residents of the U.S.

Few studies have examined barriers to accessibility of ASD services in rural areas of the contiguous U.S. from the perspective of parents and service providers, and even fewer have specifically focused on the experience of those living and working in Appalachia.^14^ The Appalachian region refers to the cultural area along the Appalachian Mountains in the eastern U.S. from western New York state to northern Alabama and Mississippi. This is a particularly relevant region on which to focus because of the significant and persistent health and economic disparities that impact this region, including ranking low compared to the rest of the nation on various health indicators, having higher rates of mortality, and having lower rates of healthcare professionals working in the region (i.e., primary care physicians, mental health providers, and specialty providers).^15^ The extant literature on the availability and accessibility of ASD-specific services in Appalachia has tended to focus on the barriers to accessing services through the public school system.^14^

As such, the barriers to accessing ASD services through community service mental health providers is less clear. The availability of mental health professionals in Appalachia is lacking, with numbers reported to be about 35% below the national average and many counties designated as health provider shortage areas.^16^,^17^ It has been suggested that cultural factors need to be considered when delivering psychological counseling and related services to individuals from this region. In particular, possibly due to a history of isolation and exploitation by others, people from the cultural Appalachian region may have a strong sense of family connectedness, autonomy, and loyalty to place, and may mistrust outside professionals.^17^

The current study attempts to address this gap in the literature by reporting the results of research with residents of Virginia’s Appalachian region regarding their needs, specifically with respect to accessing evidence-based services for ASD. Just as barriers have been identified in rural communities outside of the U.S. and in relation to public school services in Appalachian U.S., we hypothesized that similar inequities in ASD assessment and service accessibility would also exist in relation to community services obtained by rural Appalachian residents in southwest Virginia. Besides the obvious barriers related to lack of specialty providers, geographic distance, and affordability, we also hypothesized that factors specific to Appalachian culture may prevent some families from fully engaging in treatment, particularly factors related to self-reliance and mistrust of health care professionals. Quantitative and qualitative data were collected during focus groups with service providers and parents/ caregivers of children with ASD. Data are used to inform public policy recommendations.

## METHODS

### Participants

Participants were 33 service providers and 15 caregivers (Tables 1 and 2) of children with ASD from locales served by Mount Rogers Community Services (MRCS), including Smyth, Wythe, Bland, Carroll, and Grayson counties, and Galax City in Virginia. Caregivers refer to anyone acting in the parenting role for the child. Using data from 2017 (https://datausa.io/), these locales have a population of 6,788 to 31,298, with median household incomes ranging from $31,311 to $46,927, and poverty rates ranging from 8.34% to 21.2%. Similarly, the majority of our caregiver sample (66.6%) reported incomes under $40,000.

According to the U.S. Census 2017 American Community Survey data, the MRCS catchment area is comprised of 93.2% White/Caucasian residents, 2.72% Black/African American residents and 2.24% Hispanic or Latino residents. Our caregiver sample self-identified as 86.7% Caucasian and 13.3% African American. Our provider sample self-identified as 97% Caucasian and 3% Latino.

Our sampling strategy included a convenience sample of local families, providers and/or administrators recruited by flyers advertised broadly or targeted by MRCS personnel. Flyers were mailed to families of children with ASD, hung in waiting rooms of MRCS facilities and shared with agency partners such as the Department of Social Services, the Court Services Unit, Family Preservation Services (a private mental health agency), behavior therapy services and the school system. The flyer was also posted in the local newspaper, advertised to autism specific listservs and posted on social media platforms. To be eligible to participate, participants had to live or work in the MRCS catchment area and be a caregiver or service provider of a child age 11 or younger diagnosed with ASD. Based on the demographic information, this sampling strategy recruited a fairly representative sample of participants in terms of income, although with a higher rate of African American participation and no Latino participation in the caregiver group.

### Procedures

The research protocol received university IRB approval. A convergent mixed-methods approach was used to collect quantitative questionnaire data and qualitative interview data from focus groups (see Appendixes A and B). Six focus groups were conducted in March through May 2018: one for caregivers and one for providers at each of the three centrally located MRCS offices. Focus groups varied from 2 to 14 participants (*M* = 8.67 per group) and lasted from 90 to 120 minutes. They were facilitated with the assistance of graduate research assistants and research staff who led an informational presentation about ASD and intervention services for ASD. This also included informed consent followed by semi-structured group discussion and questionnaire administration. Groups were audio- and video-recorded, supplemented with written notes. For the questionnaire, all items were read aloud by research personnel while participants wrote their responses. Research assistants answered questions for individual participants as needed. Participants were not paid; however, refreshments were provided during focus groups.

### Measures

Focus group interview items (Appendix A; see Additional Files) and surveys (Appendix B; see Additional Files) were developed based on an overarching framework of the critical components that comprise the social determinants of health. Relative to rural communities, key aspects include economic stability (e.g., poverty, unemployment) and health care (e.g., access to health care and services). Therefore, the focus was on how these social factors may influence access particularly to ASD services. More specifically, items were based on the Consolidated Framework for Implementation Research (CFIR),^18^ which is an empirically based pragmatic framework for studying the implementation of an intervention (in this case, ASD services) in a particular setting (in this case rural communities). CFIR recommends studying four domains (i.e., characteristics of the intervention, outer setting, inner setting, and characteristics of individuals who will carry out the intervention).

As such, those items included related to:

1. the qualities of the services themselves (e.g., what do you think needs to be targeted in an ASD intervention to address the families’ needs?; what types of interventions do you as parents seek from providers?);
2. outer setting or characteristics of the community (e.g., what has gotten in the way of accessing services?; what community factors would prevent you from accessing services if offered?);
3. inner setting or characteristics of the participating organizations (e.g., what barriers prevent providers from offering these services?); and
4. individual provider characteristics (e.g., as a provider, are you comfortable with your level of knowledge regarding ASD?”).

Corresponding questions were included in both the interviews and surveys in order to obtain both qualitative reports and quantitative measurement.

### Data Analysis

Data were transcribed and entered in June through November 2018, and the majority of analyses were conducted from January through May 2019 with additional analyses in October 2019. Quantitative analysis consisted of descriptive data from the survey items, which included checklists on types of services received and perceived as needed, barriers to accessing or providing services, and ratings on the importance and availability of services. Caregivers reported on community use (ever used = yes/no). Both caregivers and providers reported on availability (on a scale of 1 = low to 5 = high availability), and importance (on a scale of 1 = low to 5 = high importance) of services. In terms of community-based factors, both providers and caregivers were asked to rate barriers (on a scale of 1 = very little to 5 = very much) that may prevent clients from participating in parent-training in the community, hosted by a non-local agency. Frequency counts, means, and standard deviations were provided, as appropriate.

For qualitative analysis, the audio recordings were transcribed, and strategies were used to ensure the accuracy and quality of the transcriptions.^19^ The resultant data consisted of a total of 289 double-spaced pages of verbatim transcripts of the focus group discussions. A systematic thematic analysis of the data was conducted using Pentland’s narrative process theory,^20^ which involves identifying and examining common patterns or gaps within the data, orienting them within the context of the research problem, indexing themes, and building a framework to better understand the quantitative data that was collected through the aforementioned survey. Its applicability was based on its ability to use the “surface” perceptions of a studied group (“focal actors”) as depicted through express texts to produce a better understanding of the “deep structures” informing an identified problem. This approach allowed for the identification of the needs, barriers, and strengths identified by participants, before being consensus coded by two raters.

Specifically, the focus group transcripts provided the base texts used in the narrative analysis. Each transcript served as a distinct story told by one of two identified focal actors: caregiver or provider, each with a geographic distinction. With reference to basic word frequency counts and textual similarity analysis performed in Atlas.ti, (https://atlasti.com) the two coders performed close readings of the texts as data, which they then compared. Discrepancies were rare and resolved by reference to explicit statements evident in the transcripts’ text. Because of the recognized limitation of the small sample size, the qualitative analysis was primarily used to identify themes that indicated the presence of deeper structures. The quantitative analysis then provided more understanding of the qualitative data’s description. The data were analyzed together as one dataset, in three phases, based on location. Subsequent analysis separated the provider data from the caregiver data independent of location. The final analysis compared findings for common themes between the caregiver and provider reports using the same codebook across both caregiver and provider transcripts.

## RESULTS

### Quantitative Findings

Descriptive results (Table 3; see Additional Files) indicated that caregivers had the most difficulty with accessing ASD services due to few providers available, few providers with ASD-specific knowledge or training, geographic isolation, and affordability. Only 40% of caregivers reported that, after the diagnosis, professionals provided information about available resources, while 13.3% of caregivers received no additional information. On the question “How important is it to target these following symptoms in your child with ASD/autism?,” they rated all intervention targets as high to very high in importance, with social skills rated as most important. Most providers reported that they had difficulty providing ASD services due to lack of resources and lack of ASD-specific training, and that clients’ main barriers were transportation, lack of provider resources, geographic isolation, few providers available in general, and affordability. All intervention targets were rated as high to very high in importance, with challenging behaviors rated as most important.

Table 4 (see Additional Files) provides caregiver-reports of the frequency of services used. This table also provides the mean ratings of importance and availability of services as reported by caregivers (Table 4; see Additional Files) and providers (Table 5; see Additional Files). The top services ever used, at a frequency of 53.3% or more, were: speech/language therapy, social skills training, medications, behavioral treatment, occupational therapy, and family support services. All types of services, except for special needs camps, were rated as moderate to high in importance by caregivers, ranging from a mean of 3.54 to 4.85. Most services were rated as low in availability by caregivers, ranging from a mean of 2 to 3.73. The services with both the highest ratings of importance (M ≧ 4.5) and lowest ratings of availability (M ≦ 2.5) included: social skills training, vocational training, family support services, parent training/coaching, and parent lectures/workshops.

For providers, all types of services were rated as moderate to high in importance, ranging from a mean of 3.69 to 4.91. Most services were rated as low in availability by providers, ranging from a mean of 1.52 to 3.45. The services with both the highest ratings of importance (M ≧ 4.5) and lowest ratings of availability (M ≦ 2.5) included: family support services, parent training/coaching, parent lectures/workshops, diagnostic services, professional/provider trainings, and educator trainings.

In terms of community-based factors that may prevent a family from engaging in treatment offered by a non-local agency (Table 6; see Additional Files), caregivers yielded very little to little ratings (ranging from 1.08 to 1.93), with the exception of childcare, which was rated at a mean of 3. Providers yielded moderate to high ratings (ranging from 2.13 to 4.41), with the highest barriers noted for location and ability to get to the training, lack of childcare, family issues, and service provided by “outsiders” who do not understand the community. These data indicate that caregivers perceived few community barriers preventing them from participating in treatment, except for a moderate barrier of lack of childcare. Providers perceived additional barriers related to transportation, family, and trust issues.

Qualitative findings are included as Appendix C in Additional Files.

## IMPLICATIONS

This report aimed to fill gaps in the literature by identifying needs of citizens in the rural Appalachian region of southwest Virginia with respect to accessing evidence-based ASD services from both caregiver and provider perspectives. Results indicated three primary categories of barriers: (1) *availability* of services, (2) *affordability* of services, and (3) *cultural attitudes* regarding outside professionals. Both caregivers and providers endorsed that having few providers in general and with ASD-specific training, geographic isolation, and affordability were among the top barriers to accessing ASD services. Both providers and caregivers emphasized the need for parent training and supports. Providers also emphasized the need for professional training and diagnostic resources, while caregivers emphasized the need for vocational and social skills training for their children, which highlight the unique needs that may differ across providers and caregivers. Besides highlighting the effects of extreme poverty in these rural communities, providers also focused on cultural attitudes in the Appalachian region that they have perceived may prevent families from fully engaging in programs: mistrust of “outsiders” and interpersonal respect/autonomy. These findings are consistent with prior work on barriers to ASD service utilization,^11^–^13^ educating students with ASD in rural areas,^5^,^14^ and cultural sensitivity to the Appalachian region.^17^ These findings also support the dire need for policymakers to find ways to increase availability of trained service providers and affordability of those services. There are a number of actions that may be relevant for policy makers to consider based on the results of this study.

### Availability of ASD Services

With respect to the lack of ASD providers in rural communities, policy makers may consider supporting legislation like loan forgiveness programs and service scholarships that incentivizes working in rural areas, specifically for professionals with expertise in ASD. These types of programs have been found to be effective in recruiting teachers to underserved communities when they cover tuition cost, target high-priority fields, recruit well-prepared candidates, and provide reasonable consequences if recipients do not complete service.^21^ Similarly, additional funding for graduate/medical school programs and postdoctoral/residency training programs to support the development of clinical training opportunities working with the ASD population in underserved regions may help encourage young professionals to work in rural areas. This technique has been used successfully to train and recruit physicians to work in rural communities, and as such may be generalizable to service providers who have experience working with ASD.^22^

With respect to the concerns of caregivers of children with ASD needing additional education and evidence-based services that combine child and family needs, it is recommended that policymakers consider wider coverage under support waivers. Research has indicated that by increasing the maximum amount each state allows for individuals enrolled under service waivers, and increasing the maximum number of individuals the waiver can cover, the likelihood that parents of children with ASD have to stop working is significantly reduced.^23^ Furthermore, ASD-specific evidence-based practices exist that specifically target parent and provider knowledge and needs.^24^,^25^ Therefore, increasing service waiver coverage to include such practices may help address the care-related needs of caregivers of children with ASD.

Regarding concerns of the lack of coordinated care among service providers, policy makers may consider research on the use of novel methods of integrating care. For example, electronic health record systems have been found to improve service coordination and reduce mental health care disparities.^26^

### Affordability of ASD Services

Because affordability has been identified as a barrier to accessing services for students with ASD in previous literature^14^ and for community services in the current study, it is important to minimize any additional financial burden that these families may face. As one possibility, policy makers could consider supporting autism insurance reform mandates that require coverage of evidence-based treatments. As of August of 2019, all states have enacted autism insurance reform mandates that require health insurance coverage for ASD services as a means of reducing the cost associated with these diagnoses^27^; however, there are inconsistencies amongst these mandates on a state-by-state basis, which result in difficulties accessing services for families living in certain areas of Appalachia (i.e., Tennessee).

With respect to concerns related to the cost associated with traveling to access services, policy makers may consider supporting legislation that improves access to telehealth services, which have been proposed as a potential solution to the difficulties accessing and affording services that are not readily accessible in rural areas.^5^,^13^ Unfortunately, there are several barriers to the success of telehealth services, including limited service reimbursement, difficulties accessing interstate medical licensure, and the cost of developing a telehealth service delivery infrastructure.^28^ Finding ways to address these barriers (e.g., providing increased funding for developing necessary infrastructure within rural healthcare systems) could increase access to more affordable care in geographically isolated communities. Other potential solutions to improving access could be to integrate ASD mental health and diagnostic services within already accessible service systems, such as local schools or pediatric and primary care offices.^29^

### Cultural Attitudes Toward Professionals

When considering increasing the workforce of mental health providers in rural Appalachian regions, policy makers and educators may need to consider the addition of multicultural training that is specific and sensitive to the history and needs of Appalachian residents. It has been suggested, for example, that providers need to spend more time building rapport with clients by engaging in chit-chat and pleasantries (i.e., “front porch talk”), and getting to know specific terms that are used in the region (e.g., “having nerves” to mean experiencing anxiety).^17^ Similarly, providers in our sample suggested the need to establish trust and to respect client autonomy by becoming part of the community, avoiding jargon, and treating clients without condescension. Diversity training that includes such information about cultural attitudes may help overcome some initial mistrust of professionals and facilitate better therapeutic relationships. Interestingly, the caregivers themselves did not report these cultural barriers, noting only childcare as the primary barrier to participating in treatment. It is not clear if the lack of caregiver report was due to the small sample size, or if there is a real discrepancy in how families versus providers view barriers to seeking treatment.

Despite the policy implications of these findings, there are a number of shortcomings that are important to acknowledge in hopes of directing future research efforts. While service provider participation was notable, caregiver participation was limited, as recruiting caregivers of children with disabilities in rural areas is inherently challenging. As a result, saturation determination with regard to qualitative findings is limited. However, the data obtained indicate the need for more research on the accessibility of ASD-services in rural communities that features the use of other instruments, including extensive standardized surveys. The current study also did not directly ask participants about the specific impact of the Appalachian region, but rather assessed barriers generally related to the community (e.g., attitudes toward outsiders, mental health, parenting, inconsistent transportation). Future research will need to consider additional cultural factors that are specific to this region.

This report provides suggestions for policymakers to address disparities in ASD services by paying attention to the unique barriers faced by rural citizens. Early evidence-based services for ASD are critical to the best short-term outcome and long-term prognosis. Such services need to be *available* from trained professionals and *affordable* to clients. Also, supportive services are needed to address the stress and added mental health burdens placed on caregivers and *cultural attitudes* that may interfere with treatment participation. We encourage policymakers to consider how best to embody these policy recommendations into laws or regulations that will promote ASD service equity and reduce the long-term economic and mental health burdens on families and society.

**SUMMARY BOX**

**What is already known about this topic?** Low-resource rural communities face significant challenges regarding availability and adequacy of evidence-based services; this is especially so for families impacted by a diagnosis of Autism Spectrum Disorder (ASD).

**What is added by this report?** This study supported the barriers of availability and affordability of ASD services in rural Appalachia, as well as possible cultural attitudes that may prevent engagement in treatment. The findings shed light on the need for more ASD-trained service providers, better coordination and navigation of services, addition of programs to assist with family financial and emotional stressors, and multicultural sensitivity training of service providers.

**What are the implications for public health practice, policy, and research?** Specific policy recommendations are noted in attempts to address disparities in ASD services by paying attention to the unique barriers faced by rural citizens.

**TABLE 1:** Frequency (N & %) of Caregiver Demographics

		N	Percentage
**Gender**			
Male	Caregiver	4	26.7
	Child	10	66.7
Female	Caregiver	11	73.3
	Child	5	33.3
**Child Age (years)**			
5		2	13.3
6		1	6.7
7		1	6.7
8		1	6.7
9		3	20.0
10		2	13.3
11		2	13.3
12		3	20.0
**Income Level ($)**			
Under 20K		5	33.3
20–39,999K		5	33.3
40–59,999K		1	6.7
60–79,999K		2	13.3
80–99,999K		0	0.0
≥100K		1	6.7
**Ethnicity**			
Caucasian		13	86.7
African American		2	13.3
**Focus Group Location**			
Galax City		9	60.0
Smyth County		1	6.7
Wythe & Bland Counties		5	33.3

Note: Not all participants reported income.

**TABLE 2:** Frequency (N & %) of Provider Demographics

	N	Percentage
**Gender**		
Male	4	12.1
Female	29	87.9
**Years in Profession**		
0–2	9	27.3
3–5	6	18.2
6–10	5	15.2
11–15	4	12.3
16–25	3	9.0
≥26	6	18.2
**Years of Experience with ASD Clients**		
0–2	5	15.2
3–5	7	21.2
6–10	10	30.3
11–15	4	12.1
16–25	3	9.1
≥26	4	12.1
**Ethnicity**		
Caucasian	32	97.0
Latino/Hispanic	1	3.0
**Focus Group Location**		
Galax City	13	39.4
Smyth County	11	33.3
Wythe & Bland Counties	9	27.3

## Supplementary Information















## References

[1] Masi A DeMayo MM Glozier N Guastella AJ An overview of autism spectrum disorder, heterogeneity and treatment options Neuroscience bulletin 2017 April 33 2 183 193 10.1007/s12264-017-0100-y 28213805 PMC5360849

[2] Buescher AV Cidav Z Knapp M Mandell DS Costs of autism spectrum disorder in the United Kingdom and the United States JAMA Pediatrics 2014 Aug 168 8 721 8 10.1001/jamapediatrics. 24911948

[3] Koegel LK Koegel RL Ashbaugh K Bradshaw J The importance of early identification and intervention for children with or at risk for autism spectrum disorders Int J Speech Lang Pathol 2014 Feb 16 1 50 6 10.3109/17549507.2013.861511. 24328352

[4] Smith T Iadarola S Evidence base update for autism spectrum disorder J Clin Child Adolesc Psychol 2015 44 6 897 922 10.1080/15374416.2015.1077448. 26430947

[5] Antezana L Scarpa A Valdespino A Albright J Richey JA Rural trends in diagnosis and services for autism spectrum disorder Front Psychol 2017 Apr 20 8 590 10.3389/fpsyg.2017.00590. 28473784 PMC5397491

[6] Rhoades RA Scarpa A Salley B The importance of physician knowledge of autism spectrum disorder: results of a parent survey BMC Pediatr 2007 Nov 20 7 37 18021459 10.1186/1471-2431-7-37 PMC2235850

[7] Khowaja MK Hazzard AP Robins DL Sociodemographic barriers to early detection of autism: screening and evaluation using the M-CHAT, M-CHAT-R, and follow-up J Autism Dev Disord 2015 Jun 45 6 1797 808 10.1007/s10803-014-2339-8. 25488122 PMC4442731

[8] Mello MP Goldman SE Urbano RC Hodapp RM Services for children with autism spectrum disorder: Comparing rural and non-rural communities Education and Training in Autism and Developmental Disabilities 2016 Dec 1 355 65

[9] Murphy MA Ruble LA A comparative study of rurality and urbanicity on access to and satisfaction with services for children with autism spectrum disorders Rural Special Education Quarterly 2012 Sep 31 3 3 11 10.1177/875687051203100302

[10] Scarpa A White SW Attwood T CBT for children and adolescents with high-functioning autism spectrum disorders Guilford Press 2013 Jul 25

[11] Ashburner J Vickerstaff S Beetge J Copley J Remote versus face-to-face delivery of early intervention programs for children with autism spectrum disorders: Perceptions of rural families and service providers Research in Autism Spectrum Disorders 2016 Mar 1 23 1 4 10.1016/j.rasd.2015.11.011

[12] Hussain R Tait K Parental perceptions of information needs and service provision for children with developmental disabilities in rural Australia Disabil Rehabil 2015 37 18 1609 16 10.3109/09638288.2014.972586. 25332090

[13] Iacono T Dissanayake C Trembath D Hudry K Erickson S Spong J Family and practitioner perspectives on telehealth for services to young children with autism The Promise of New Technologies in an Age of New Health Challenges. Stud Health Technol Inform 2016 231 63 73 27782017

[14] Azano AP Tackett ME Perceptions of Teachers and Parents on the Educational Experiences of Students with Autism in a Remote Rural Community The Rural Educator 2017 Dec 11 38 3 10.35608/ruraled.v38i3.219

[15] Marshall JL Thomas L Lane NM Holmes GM Arcury TA Randolph R Silberman P Holding W Villamil L Thomas S Lane M Latus J Rodgers J Ivey K Creating a culture of health in Appalachia: Disparities and Bright Spots Appalachian Regional Commission 2017

[16] Heisler EJ The mental health workforce: A primer (Congressional Research Services 7, 7500 2018 Retrieved from https://fas.org/sgp/crs/misc/R43255.pdf

[17] Thomas ME Brossoie N Appalachia mental healthcare: An interpretative phenomenological analysis study to identify training program needs Journal of Rural Mental Health 2019 Apr 43 2–3 91 10.1037/rmh0000116

[18] Damschroder LJ Aron DC Keith RE Kirsh SR Alexander JA Lowery JC Fostering implementation of health services research findings into practice: a consolidated framework for advancing implementation science Implementation science 2009 Dec 4 1 50 10.1186/1748-5908-4-50 19664226 PMC2736161

[19] Holstein J Gubrium JF Inside interviewing: New lenses, new concerns Sage 2003

[20] Pentland BT Building process theory with narrative: From description to explanation Academy of management Review 1999 Oct 1 24 4 711 24 10.2307/259350

[21] Podolsky A Kini T How effective are loan forgiveness and service scholarships for recruiting teachers 2016 Palo Alto, CA Learning Policy Institute 42 48

[22] Mareck DG Federal and state initiatives to recruit physicians to rural areas AMA Journal of Ethics 2011 May 1 13 5 304 9 10.1001/virtualmentor.2011.13.5.pfor1-1105. 23131362

[23] Leslie DL Iskandarani K Velott DL Stein BD Mandell DS Agbese E Dick AW Medicaid waivers targeting children with autism spectrum disorder reduce the need for parents to stop working Health Aff (Millwood) 2017 Feb 1 36 2 282 288 10.1377/hlthaff.2016.1136. 28167717 PMC5819340

[24] Ruble LA McGrew JH Toland MD Dalrymple NJ Jung LA A randomized controlled trial of COMPASS web-based and face-to-face teacher coaching in autism J Consult Clin Psychol 2013 Jun 81 3 566 72 10.1037/a0032003. 23438314 PMC3744829

[25] Kuravackel GM Ruble LA Reese RJ Ables AP Rodgers AD Toland MD Compass for hope: Evaluating the effectiveness of a parent training and support program for children with ASD J Autism Dev Disord 2018 Feb 48 2 404 416 10.1007/s10803-017-3333-8. 29022130

[26] McGregor B Mack D Wrenn G Shim RS Holden K Satcher D Improving service coordination and reducing mental health disparities through adoption of electronic health records Psychiatr Serv 2015 Sep 66 9 985 7 10.1176/appi.ps.201400095. 25975885 PMC4558322

[27] Cernius A No Imbecile at All: How California Won the Autism Insurance Reform Battle, and Why Its Model Should be Replecated in Other States Harv L & Pol’y Rev 2016 10 565

[28] Weinstein RS Lopez AM Joseph BA Erps KA Holcomb M Barker GP Krupinski EA Telemedicine, telehealth, and mobile health applications that work: opportunities and barriers Am J Med 2014 Mar 127 3 183 7 10.1016/j.amjmed.2013.09.032. 24384059

[29] Centers for Disease Control and Prevention Providing access to mental health services for children in rural areas Morbidity and mortality weekly report: MMWR Atlanta, Ga U.S. Dept. of Health, Education, and Welfare, Public Health Service Center for Disease Control https://www.cdc.gov/ruralhealth/child-health/policybrief.html Accessed October 18, 2019

