# Supplementary material for: Access to Autism Spectrum Disorder Services for Rural Appalachian Citizens
Source: J Appalach Health. 2020 Jan 26;2(1):25–40. doi: 10.13023/jah.0201.04 (PMC9138840; doi:10.13023/jah.0201.04)
Supplement: Supplementary file 1 [file 1027-AppendixA-Scarpa-2.1.4.pdf]

# Appendix A

## Focus Group Questions: Caregivers

<u>Service Availability and Accessibility</u>

1. What types of interventions do you as parents seek from providers? Probe for:
    a. Communication (e.g., speech, language, comprehension),
    b. Social engagement (eye contact, reciprocity, relationship quality);
    c. Social skills (making and keeping friends),
    d. Behavior interventions (aggression, anger, following rules),
    e. Other? (daily living and self-help skills, anxiety, transition to middle school)
2. What services have you received or used in your community?
    a. Trainings/workshops?
    b. Lectures/speakers?
    c. Autism support groups/Advocacy groups?
    d. Parent training for treating the child with ASD?
        i. What kind of parent training?
        ii. What content was included?
    e. Other?
3. In general, were these services helpful for you? Why or why not?
4. What services do you still need, but are not able to get locally (meaning not easily accessible to you)?
5. What has gotten in the way of accessing services?
    a. Few providers available in general?
    b. Few providers with autism specific knowledge or training available?
    c. Lack of resources for providers.
    d. Difficulty with transition from early intervention to educational system?
    e. Travel/transportation issues?
    f. Geographic location?
    g. Affordability?
    h. Disability?
    i. Lack of childcare?
    j. Other?
6. What could help with access to these services?

<u>Community Factors</u>

7. What general advice do you have for us when providing services in your community?
8. What barriers might there be for parents to participate in and complete a parent training program?
    i. Regional/societal attitudes towards seeking help?
    ii. Perceptions of being criticized or judged as a parent?
    iii. Confidentiality/privacy?
    iv. Attitudes towards mental health or autism?
    v. Family issues?
    vi. Lack of childcare?

vii. Service provided by “outsiders”?
viii. Religion or specific values and beliefs about parenting?
ix. Health concerns or issues?
x. Location?
xi. Other?

**Focus Group Questions: Providers**

<u>Service Availability and Accessibility</u>

1. As providers, what do you think needs to be targeted in an ASD intervention to address these families’ needs?
    b. Communication (e.g., speech, language, comprehension),
    c. Social engagement (eye contact, reciprocity, relationship quality);
    d. Social skills (making and keeping friends),
    e. Behavior interventions (aggression, anger, following rules),
    f. Other? (daily living and self-help skills, anxiety, transition to middle school)
2. What services are still needed, but clients are not able to get locally (meaning not easily accessible)?
    a. What are barriers preventing family access to these services?
        i. Few providers available in general?
        ii. Few providers with autism specific knowledge or training available?
        iii. Lack of resources for providers?
        iv. Difficulty with transition from early intervention to educational system?
        v. Travel/transportation issues?
        vi. Geographic location?
        vii. Affordability?
        viii. Disability?
        ix. Lack of childcare?
        x. Other?
    g. What are barriers preventing you from offering these services?
        i. Lack of autism specific knowledge?
        ii. Travel/transportation issues?
        iii. Geographic location?
        iv. Lack of appropriate training?
        v. Lack of support from supervisor/leadership?
        vi. Lack of resources (supplies, manuals, training materials)
        vii. Implementation cost?
        viii. Other?
    h. What could help with access to these services?
3. Are you comfortable with your level of knowledge regarding ASD? Why or why not?
    a. If not, what types of professional development would be helpful to you/staff related to ASD?

Community Factors

4. What general advice do you have for us when providing services in the community in which you work?
5. What barriers might there be for parents to participate in and complete a parent training program?
    i. Regional/societal attitudes towards seeking help?
    ii. Perceptions of being criticized or judged as a parent?
    iii. Confidentiality/privacy?
    iv. Attitudes towards mental health or autism?
    v. Family issues?
    vi. Lack of childcare?
    vii. Service provided by “outsiders”?
    viii. Religion or specific values and beliefs about parenting?
    ix. Health concerns or issues?
    x. Location?
    xi. Other?
