# Supplementary material for: Access to Autism Spectrum Disorder Services for Rural Appalachian Citizens
Source: J Appalach Health. 2020 Jan 26;2(1):25–40. doi: 10.13023/jah.0201.04 (PMC9138840; doi:10.13023/jah.0201.04)
Supplement: Supplementary file 2 [file 1027-AppendixB-Scarpa-2.1.4.pdf]

Appendix B

Focus Group Rating Scales: Caregivers

These questions refer specifically to your child with ASD/autism. If more than one, choose the child with ASD/autism that is the youngest.

1. How old is your child with ASD/autism?
   _________ years, _________ months

2. What is your child's gender?
   ___ Male
   ___ Female
   ___ Another

3. What is your child's current ASD/autism diagnosis?
   ___ Autism Spectrum Disorder
   ___ Autistic Disorder
   ___ Asperger's Disorder
   ___ Pervasive Developmental Disorder – Not Otherwise Specified (PDD-NOS)
   ___ Other (please specify __________________________)

4. How old was your child when he/she got this ASD/autism diagnosis?
   ___ years, ___ months

5. What type of professional diagnosed your child with ASD/autism?
   ___ Developmental Pediatrician
   ___ Psychologist
   ___ Neurologist
   ___ Primary Care Physician
   ___ Psychiatrist
   ___ Other (please specify _____________________________________)

6. What did that professional do after diagnosing your child? Check all that apply.
   ___ Provided no additional information
   ___ Gave you information about available resources
   ___ Gave you literature on Autism
   ___ Spent time talking with you about Autism
   ___ Referred you to an Autism specialist
   ___ Referred you to a support group
   ___ Advised on educational programs
   ___ Advised on medical problems
   ___ Other (please specify _____________________________________

Service Availability and Accessibility

7. What makes it hard for your family to access ASD services?
   ___Few providers available in general
   ___Few providers with ASD knowledge or training available
   ___Travel/Transportation issues
   ___Geographic location or isolation
   ___Affordability
   ___Disability
   ___Lack of childcare
   ___Other? (please specify) _______________

Circle the number that best reflects your opinion, using the following scale:

Very Low	Low	Moderate	High	Very High
**1**	**2**	**3**	**4**	**5**

8. How important is it to target these following symptoms in your child with ASD/autism?

Communication (understanding or expressing verbal language; using another communication device)	1 2 3 4 5
Social Skills (learning to make and keep friends, conversations, dealing with conflict, etc)	1 2 3 4 5
Social Engagement (reciprocity, eye contact, sharing enjoyment, etc.)	1 2 3 4 5
Challenging Behavior (aggression, tantrums, repetitive behaviors)	1 2 3 4 5
Other (please specify)	1 2 3 4 5

9. Which of these ASD/autism services have you or your child **used** before? (Check all that apply)
   ___ Behavioral treatment (such as Applied Behavior Analysis, Pivotal Response Training, Discrete Trial Training, or Positive Behavior Supports)
   ___ Social skills training
   ___ Speech/language therapy
   ___ Early intervention
   ___ Music, Dance, Art, or Drama Therapy
   ___ Sensory integration therapy
   ___ Occupational therapy
   ___ Physical therapy
   ___ Vocational training
   ___ Animal therapy
   ___ Dietary interventions (such as Casein-free/Gluten free diets, Fatty acids, Feingold diet, Alkaline shots, Magnesium, Vitamin supplements)
   ___ Respite care
   ___ Family support services
   ___ Parent training/coaching
   ___ Parent lectures/workshops
   ___ Medications (such as SSRIs, stimulants, antipsychotics, etc.)
   ___ Diagnostic services
   ___ Web-based resource network
   ___ Special needs camps
   ___ Other (please specify) ______________________

10. How **available** are these services in your county, whether you have used them or not? Use the following scale:

Very Low Availability	Low	Moderate	High	Very High Availability
1	2	3	4	5

___ Behavioral treatment (such as Applied Behavior Analysis, Pivotal Response Training, Discrete Trial Training, or Positive Behavior Supports)
___ Social skills training
___ Speech/language therapy
___ Early intervention
___ Music, Dance, Art, or Drama Therapy
___ Sensory integration therapy
___ Occupational therapy
___ Physical therapy
___ Vocational training
___ Animal therapy
___ Dietary interventions (such as Casein-free/Gluten free diets, Fatty acids, Feingold diet, Alkaline shots, Magnesium, Vitamin supplements)
___ Respite care
___ Family support services
___ Parent training/coaching
___ Parent lectures/workshops
___ Medications (such as SSRIs, stimulants, antipsychotics, etc.)
___ Diagnostic services
___ Web-based resource network
___ Special needs camps
___ Other (please specify) ______________________

11. How **important** is it for these services to be offered in your county, whether you have used them or not? Use the following scale:

Very Low Importance	Low	Moderate	High	Very High Importance
1	2	3	4	5

___ Behavioral treatment (such as Applied Behavior Analysis, Pivotal Response Training, Discrete Trial Training, or Positive Behavior Supports)
___ Social skills training
___ Speech/language therapy
___ Early intervention
___ Music, Dance, Art, or Drama Therapy
___ Sensory integration therapy
___ Occupational therapy
___ Physical therapy
___ Vocational training
___ Animal therapy
___ Dietary interventions (such as Casein-free/Gluten free diets, Fatty acids, Feingold diet, Alkaline shots, Magnesium, Vitamin supplements)
___ Respite care
___ Family support services

___ Parent training/coaching
___ Parent lectures/workshops
___ Medications (such as SSRIs, stimulants, antipsychotics, etc.)
___ Diagnostic services
___ Web-based resource network
___ Special needs camps
___ Professional/provider trainings
___ Educator trainings
___ Other (please specify) _______________________

12. If Virginia Tech offered parent training in your community, would any of these things prevent you from participating? Use the following scale.

Very Little	Little	Moderate	A lot	Very Much
1	2	3	4	5

___ Your attitude towards seeking help?
___ Your sense of being criticized or judged as a parent?
___ Concern about your privacy?
___ Your attitudes towards mental health or autism?
___ Family issues?
___ Lack of childcare?
___ Service provided by "outsiders" who do not understand your community?
___ Your religion or specific values and beliefs about parenting?
___ Your health concerns or issues?
___ Your location and ability to get to the training?
___ Other? (please specify) ________________

Focus Group Rating Scales: Providers

Service Availability and Accessibility

1. What makes it hard for your clients to access ASD services?
___ Few providers available in general
___ Few providers with ASD knowledge or training available
___ Lack of resources for providers
___ Difficulty with transition from early intervention to educational system
___ Travel/Transportation issues
___Geographic location or isolation
___Affordability
___Disability
___Lack of childcare
___Other? (please specify) ____________________

2. What makes it hard for you to offer or provide ASD services?
___ Lack of autism specific knowledge?
___ Travel/transportation issues?
___ Geographic location?
___ Lack of appropriate training?
___ Lack of support from supervisor/leadership?
___ Lack of resources (supplies, manuals, training materials)

___ Implementation cost?
___ Other? (please specify) ______________________

Circle the number that best reflects your opinion, using the following scale:

**Very Low**	**Low**	**Moderate**	**High**	**Very High**
**1**	**2**	**3**	**4**	**5**

3. How important is it to target these following symptoms in your child clients with ASD/autism?

Communication (understanding or expressing verbal language; using another communication device)	1 2 3 4 5
Social Skills (learning to make and keep friends, conversations, dealing with conflict, etc)	1 2 3 4 5
Social Engagement (reciprocity, eye contact, sharing enjoyment, etc.)	1 2 3 4 5
Challenging Behavior (aggression, tantrums, repetitive behaviors)	1 2 3 4 5
Other (please specify)	1 2 3 4 5

7. Which of these ASD/autism services have you **offered or provided** before? (Check all that apply)
   ___ Behavioral treatment (such as Applied Behavior Analysis, Pivotal Response Training, Discrete Trial Training, or Positive Behavior Supports)
   ___ Social skills training
   ___ Speech/language therapy
   ___ Early intervention
   ___ Music, Dance, Art, or Drama Therapy
   ___ Sensory integration therapy
   ___ Occupational therapy
   ___ Physical therapy
   ___ Vocational training
   ___ Animal therapy
   ___ Dietary interventions (such as Casein-free/Gluten free diets, Fatty acids, Feingold diet, Alkaline shots, Magnesium, Vitamin supplements)
   ___ Respite care
   ___ Family support services
   ___ Parent training/coaching
   ___ Parent lectures/workshops
   ___ Medications (such as SSRIs, stimulants, antipsychotics, etc.)
   ___ Diagnostic services
   ___ Web-based resource network
   ___ Special needs camps
   ___ Professional/provider trainings
   ___ Educator trainings
   ___ Other (please specify) ______________________

8. How **available** are these services in your county, whether you have offered them or not? Use the following scale:

Very Low Availability	Low	Moderate	High	Very High Availability
1	2	3	4	5

___ Behavioral treatment (such as Applied Behavior Analysis, Pivotal Response Training, Discrete Trial Training, or Positive Behavior Supports)
___ Social skills training
___ Speech/language therapy
___ Early intervention
___ Music, Dance, Art, or Drama Therapy
___ Sensory integration therapy
___ Occupational therapy
___ Physical therapy
___ Vocational training
___ Animal therapy
___ Dietary interventions (such as Casein-free/Gluten free diets, Fatty acids, Feingold diet, Alkaline shots, Magnesium, Vitamin supplements)
___ Respite care
___ Family support services
___ Parent training/coaching
___ Parent lectures/workshops
___ Medications (such as SSRIs, stimulants, antipsychotics, etc.)
___ Diagnostic services
___ Web-based resource network
___ Special needs camps
___ Professional/provider trainings
___ Educator trainings
___ Other (please specify) ________________________

9. How **important** is it for these services to be offered in your county, whether you have offered them or not? Use the following scale:

Very Low Importance	Low	Moderate	High	Very High Importance
1	2	3	4	5

___ Behavioral treatment (such as Applied Behavior Analysis, Pivotal Response Training, Discrete Trial Training, or Positive Behavior Supports)
___ Social skills training
___ Speech/language therapy
___ Early intervention
___ Music, Dance, Art, or Drama Therapy
___ Sensory integration therapy
___ Occupational therapy
___ Physical therapy
___ Vocational training
___ Animal therapy
___ Dietary interventions (such as Casein-free/Gluten free diets, Fatty acids, Feingold diet, Alkaline shots, Magnesium, Vitamin supplements)
___ Respite care
___ Family support services
___ Parent training/coaching
___ Parent lectures/workshops
___ Medications (such as SSRIs, stimulants, antipsychotics, etc.)
___ Diagnostic services

___ Web-based resource network
___ Special needs camps
___ Professional/provider trainings
___ Educator trainings
___ Other (please specify) ______________________

10. If Virginia Tech offered parent training in your community, would any of these things prevent your clients from participating? Use the following scale.

Very Little	Little	Moderate	A lot	Very Much
1	2	3	4	5

___ Attitude towards seeking help?
___ Sense of being criticized or judged as a parent?
___ Concern about their privacy?
___ Attitudes towards mental health or autism?
___ Family issues?
___ Lack of childcare?
___ Service provided by “outsiders” who do not understand the community?
___ Religion or specific values and beliefs about parenting?
___ Health concerns or issues?
___ Location and ability to get to the training?
___ Other? (please specify) _______________
