# Supplementary material for: Access to Autism Spectrum Disorder Services for Rural Appalachian Citizens
Source: J Appalach Health. 2020 Jan 26;2(1):25–40. doi: 10.13023/jah.0201.04 (PMC9138840; doi:10.13023/jah.0201.04)
Supplement: Supplementary file 3 [file 1027-AppendixC-Scarpa-2.1.4.pdf]

Appendix C

Qualitative Findings

Emergent themes from focus groups indicated that the region's needs for ASD services are multi-faceted, but three major barriers need to be addressed to increase access to appropriate evidence-based care: *availability, affordability, and cultural attitudes*.

Theme 1 (Availability):

a. The region lacks sufficient resources for diagnosis and treatment. As one provider reported,
"There's just not enough of us, to me that's the biggest issue. ... We have some of the most profound [autistic] students in counseling, but the most we can get them is every other week. And it's just not enough and it's no one's fault, it's just not enough."

b. The region lacks sufficient training and personal support resources for caregivers, leaving them overwhelmed and unable to engage in treatment. As one provider observed,
"From day one [of] being a parent, you start off exhausted.... So, they [parents] aren't following through because they don't have it within them to follow through. They've already reached the point that they can't do anything beyond that."

c. The region lacks coordination among medical providers, public school providers, and other community providers. As noted by one caregiver, for example,
"I don't really have a clear understanding of even what's available here."

d. In addition to stories that highlight poverty (see below), the transcripts reveal pervasive themes of isolation, both geographic and social, in these rural, mountainous areas.

> *"I am one person that covers our entire region, so I have Smyth, Wythe, Bland, Carroll, Galax, and Grayson."*
>
> *"Yeah, but it's a huge county, you know? It's a big area and I mean, it's very rural and, I mean, it's hard to get families here for services and it's hard to get providers to them."*

"And too, when you get in a lot of these places there is no phone coverage except for land lines and there is not Internet and you couldn't, you know, you would have a hard time taking a service to them where they are."

Theme 2 (Affordability):

a. Caregivers lack resources to pay for services, so they struggle with choosing to pay for physical needs (e.g., diapers, household heat) versus child behavior and mental health treatment (e.g., aggression, anxiety, trauma). A provider noted the following,
"*I have a little girl who's not potty-trained and her mom said she no longer gets Medicaid because she's not eligible and she has to buy the Pampers, and they're expensive… Right now, in this parent's mind is, does she look at therapy or does she look at diapers for my child?... It seems like with my parents, there's never enough to do both."*

b. The region lacks resources to fund local providers or programs, leading families to travel long distances or have lengthy wait times to get services. For example, caregivers noted,
"I was hearing people the other day talking about driving 3 or 4 hours and I'm thinking he would never make it. He does not ride in the car well … by the time we drove 3 hours and tried to do therapy he would be a mess. And then tried to drive home?"

"I wish we had something closer in the area…It's a battle to get up there. It's a long wait. We missed our appointment for snow back in December or November and couldn't go, and our next appointment's July...and there's nothing in our area, there's nothing closer."

"The day camp that my son goes to on Saturday; they can't do nothin' no more because they have no funding for it."

"The same goes with the school systems. Most of the time they turn you down and tell you they can't help you because they don't have the money to get the provider they need to get you the help you need."

Theme 3 (Cultural Attitudes):

a. Community-based factors related to autonomy and fear of outsiders may prevent families from seeking and fully engaging in programs that may be offered by external providers. Providers are aware that outsiders are frequently distrusted

and sometimes viewed as introducing risk to the family, as noted by the following quotes:

> *"They just don't want people in their homes, period."*

> *"I think, sometimes too, people have things they want to hide, We have a lot of substance abuse issues in our area . . . and . . . and a lot of parents are struggling with those kinds of issues. We have a lot of impoverished situations and we see some pretty poor environmental conditions for kids and . . . They don't want people to . . . get in their business and know about those things because of the fear that . . . that we're coming in to try to take their children away or do something that's going to be harmful."*

> *"I think it's their culture. In southwest Virginia, that is a true cultural norm. I mean, even looking back into, um, right after the Civil War. It's the cultural norm. You don't let people into your business. Your business is your business and you take care of your own and you take care of your own family. And if an outsider comes in, that's an outsider and you don't trust them because they're just there to steal from you and take from you and you get rid of them and that's a cultural norm especially in southwest Virginia. Outsiders are bad."*

b. Loyalty to place and interpersonal respect are important factors to establishing trust. Providers suggested themes like avoiding jargon, big words, or "talking down" to clients from the region, and finding ways to integrate with the community. For example:

> *". . . speak common to them. They don't like it when people come in and talk very, very educated to them because it makes them feel .... feel inferior and they don't like it and it causes a lot of rift."*

> *"I mean they can be super intelligent. They really can. I mean they can be super, scary, crazy smart but they do not want you to come in and talk down to them . . ."*

> *"I think that to show you become a part of this—a part of this community.... you get out there and become a part of it and eventually they start to know you and they'll accept you more. That's . . . how you become part of the community."*
