# Supplementary material for: Access to Autism Spectrum Disorder Services for Rural Appalachian Citizens
Source: J Appalach Health. 2020 Jan 26;2(1):25–40. doi: 10.13023/jah.0201.04 (PMC9138840; doi:10.13023/jah.0201.04)
Supplement: Supplementary file 4 [file 1027-T3-Scarpa-2.1.4.pdf]

TABLE 3: Frequency (%) of items endorsed for Information given by professionals and Barriers to accessing or providing services; Means (SDs) for Importance of intervention targets rated on a 1 (very low) to 5 (very high) scale

Item	Caregivers	Providers
**Information given by professionals following**	40.0%	n/a
**diagnosis:**	33.3%	n/a
Gave information about available resources	33.3%	n/a
Discussed ASD	33.3%	n/a
Referred to ASD specialist	26.7%	n/a
Referred to support group	26.7%	n/a
Provided literature on ASD	13.3%	n/a
Advised on education program	13.3%	
Advised on medical problems		
Provided no additional information		
**Barriers to accessing or providing ASD services:**		
Few providers available in general	66.7%	69.7%
Few providers with ASD knowledge or	66.7%	81.8%
training	40.0%	63.6%
Affordability	40.0%	72.7%
Geographic location/isolation	26.7%	78.8%
Travel/transportation issues	20.0%	42.4%
Lack of childcare	6.7%	30.3%
Disability	n/a	75.8%
Lack of resources for providers		
**Importance of intervention target**:		
Social Skills	4.80	4.68
Communication	(0.41)	(.60)
Social Engagement	4.20	4.68
Challenging Behaviors	(1.21)	(.65)
	4.47	4.49
	(0.64)	(.62)
	4.20	4.79
	(1.15)	(.42)
