# Supplementary material for: Access to Autism Spectrum Disorder Services for Rural Appalachian Citizens
Source: J Appalach Health. 2020 Jan 26;2(1):25–40. doi: 10.13023/jah.0201.04 (PMC9138840; doi:10.13023/jah.0201.04)
Supplement: Supplementary file 5 [file 1027-T4-Scarpa-2.1.4.pdf]

TABLE 4. Caregiver-Report on Frequency of Use (% of sample endorsing yes), and Availability/Importance of Services; Means (SDs) for Availability / Importance of Services rated on a 1(very low) to 5(very high) scale

Intervention Type	***Services Used***	***Availability M (SD)***	***Importance M (SD)***
Behavioral treatment	53.3%*	2.58 (1.56)	4.57 (1.09)
**Social skills training**	60.0 %*	**2.08** (1.31)	**4.85** (0.38)
Speech/language therapy	66.7%*	3.73 (1.39)	4.79 (0.43)
Early intervention	26.7%	3.2 (1.4)	4.5 (1.09)
Music, dance, art, drama therapy	13.3%	2 (1.25)	4.36 (1.08)
Sensory integration therapy	40.0%	2.18 (1.47)	4.43 (1.15)
Occupational therapy	53.3%*	3.5 (1.4)	4.79 (0.43)
Physical therapy	33.3%	3.55 (1.44)	4.57 (0.85)
**Vocational training**	6.7%	**2.22** (1.3)	**4.64** (0.63)
Animal therapy	6.7%	2.09 (1.58)	4.15 (1.46)
Dietary interventions	0.0%	1.7 (1.25)	3.54 (1.71)
Respite care	20.0%	2.91 (1.87)	4.21 (1.31)
**Family support services**	53.3%*	**2.5** (1.73)	**4.57** (1.09)
**Parent training/coaching**	33.3%	**2.25** (1.42)	**4.57** (1.09)
**Parent lectures/workshops**	26.7%	**2.17** (1.47)	**4.79** (0.43)
Medications	60.0%*	3.58 (1.68)	3.62 (1.61)
Diagnostic services	13.3%	2.36 (1.43)	3.79 (1.63)
Web-based resource network	20.0%	2.45 (1.44)	3.86 (1.61)
Special needs camps	13.3%	2.27 (1.79)	0 (0)

* = Services ever used by more than 50% of the sample.

Note: Bolded items refer to services rated as **both** highest in importance (Mean ≧ 4.5) and lowest in availability (Mean ≦ 2.5).
