# Supplementary material for: Access to Autism Spectrum Disorder Services for Rural Appalachian Citizens
Source: J Appalach Health. 2020 Jan 26;2(1):25–40. doi: 10.13023/jah.0201.04 (PMC9138840; doi:10.13023/jah.0201.04)
Supplement: Supplementary file 6 [file 1027-T5-Scarpa-2.1.4.pdf]

TABLE 5. Provider-Report on Availability/Importance of Services; Means (SDs) for Availability/Importance of Services rated on a 1(very low) to 5(very high) scale

Intervention Type	***Availability M (SD)***	***Importance M (SD)***
Behavioral treatment	2.72 (1.11)	4.76 (0.44)
Social skills training	2.69 (0.93)	4.91 (0.29)
Speech/language therapy	3.37 (0.93)	4.82 (0.53)
Early intervention	3.45 (0.96)	4.79 (0.55)
Music, dance, art, drama therapy	1.63 (0.79)	3.84 (1.02)
Sensory integration therapy	1.90 (1.01)	4.42 (0.75)
Occupational therapy	3.27 (1.01)	4.64 (0.60)
Physical therapy	3.42 (0.94)	4.70 (0.47)
Vocational training	2.45 (1.06)	4.44 (0.84)
Animal therapy	1.74 (0.68)	3.69 (1.03)
Dietary interventions	1.67 (0.71)	3.91 (1.06)
Respite care	1.90 (0.87)	4.16 (1.05)
**Family support services**	**2.22 (0.79)**	**4.84 (0.37)**
**Parent training/coaching**	**2.31 (0.86)**	**4.81 (0.40)**
**Parent lectures/workshops**	**1.74 (0.73)**	**4.72 (0.52)**
Medications	3.19 (1.05)	4.28 (0.92)
**Diagnostic services**	**2.35 (1.20)**	**4.56 (0.67)**
Web-based resource network	1.93 (1.03)	3.81 (1.15)
Special needs camps	1.52 (0.63)	4.34 (0.83)
**Professional/provider trainings**	**2.03 (0.91)**	**4.66 (0.65)**
**Educator trainings**	**1.93 (0.94)**	**4.75 (0.62)**

Note: Bolded items refer to services rated as **both** highest in importance (Mean ≧ 4.5) and lowest in availability (Mean ≦ 2.5).
