# Supplementary material for: Access to Autism Spectrum Disorder Services for Rural Appalachian Citizens
Source: J Appalach Health. 2020 Jan 26;2(1):25–40. doi: 10.13023/jah.0201.04 (PMC9138840; doi:10.13023/jah.0201.04)
Supplement: Supplementary file 7 [file 1027-T6-Scarpa-2.1.4.pdf]

TABLE 6. Caregiver- and Provider-Reported Community Barriers to Participating in a Training Hosted by a Non-local Agency; Means (SDs) for Caregiver / Provider rated on a 1(very low) to 5(very high) scale

**Barriers**	*Caregiver M (SD)*	*Provider M (SD)*
Attitudes towards seeking help	1.31 (1.11)	3.2 (1.02)
Sense of being criticized or judged as a parent	1.39 (0.65)	3.15 (0.91)
Concerns about privacy	1.08 (0.28)	2.70 (1.40)
Attitudes towards mental health or ASD	1.08 (0.28)	2.88 (1.01)
Family Issues	1.31 (0.63)	3.71 (1.07)
Lack of childcare	3 (1.8)	4.16 (1.02)
Service provided by "outsiders" who don't understand your community	1.85 (1.14)	3.47 (1.05)
Religion or specific values/beliefs about parenting	1.15 (0.55)	2.13 (1.09)
Health concerns or issues	1.54 (1.2)	2.48 (1.12)
Location and ability to get to the training	1.93 (1.44)	4.41 (0.71)
